# Myelodysplastic syndromes: A primary care perspective

**DOI:** 10.4102/safp.v67i1.6118

**Published:** 2025-07-14

**Authors:** Keshena Naidoo, Sharlene Parasnath

**Affiliations:** 1 Department of Family Medicine, College of Health Sciences, University of KwaZulu-Natal, Durban, South Africa; 2 Department of Clinical Haematology, College of Health Sciences, University of KwaZulu-Natal, Durban, South Africa

**Keywords:** myelodysplastic syndrome, blood disorders, stem cell transplant, neoplasm, haematological disorders

## Abstract

Myelodysplastic syndromes (MDS) are a group of blood disorders affecting the bone marrow resulting in cytopenia, blood cell dysplasia and an increased risk of progressing to acute myeloid leukaemia (AML). Myelodysplastic syndromes are more common in individuals older than 60 years, and those who have undergone radiation or chemotherapy in the past. Patients may be asymptomatic in the initial stages and can later develop fatigue, dyspnoea, frequent infections, petechiae, bruising and bleeding. Patients with persistent cytopenia (>6 months) should be investigated further and referred to a haematologist if at high risk for MDS. The definitive diagnostic test for MDS is a bone marrow biopsy. Individuals with lower-risk MDS can be managed with blood transfusions, erythropoiesis stimulating agents, growth factors and lenalidomide (an immunomodulatory drug). Higher risk MDS patients have a median survival of less than three years, with stem cell transplant as the only cure. However, less than 10% of MDS patients receive this treatment because of the scarcity of donors. Primary care providers should also be aware of long-term side effects following a stem cell transplant. This article aims to increase awareness of MDS and stem cell transplants.

## Background

Myelodysplastic syndromes (MDS), now classified as neoplasms by the World Health Organization, are a group of blood disorders that affect the bone marrow.^1^ These disorders occur when the bone marrow produces abnormal blood cells that fail to mature into healthy blood cells. Myelodysplastic syndromes are characterised by refractory cytopenias (anaemia, thrombocytopaenia and neutropenia) and progression to acute myeloid leukaemia (AML) in a third of individuals.^2,3,4^

Primary care providers may be the first point of contact for patients presenting with symptoms of MDS. They should therefore be familiar with MDS and consider further investigation of patients with cytopenia. Early identification and appropriate management or referral can improve patient outcomes and reduce the risk of progression to AML.

The incidence of MDS is about four to five cases per 100 000 people per year (more common in males), increasing to 40–50 per 100 000 in people older than 70 years.^5^ The cause is unknown in most cases but about 15% of cases occur after chemotherapy or radiotherapy for cancer.^6^ Other suspected factors include tobacco smoking and occupational exposure to solvents or agricultural chemicals.^2^ Autoimmune diseases (such as hypothyroidism, thyroiditis, ulcerative colitis, rheumatoid arthritis and systemic lupus erythematosus [SLE]) have also been associated with the development of MDS. The pathophysiology of MDS is thought to involve cytogenetic changes and/or gene mutations with widespread gene hypermethylation in advanced disease.^2^

## Clinical features

Patients with MDS can be asymptomatic in the early stages with only an incidental finding of cytopenia in a routine blood test. The most common cytopenia associated with MDS is anaemia, found in 80% – 85% of patients. Anaemia associated with MDS is usually macrocytic or normocytic.^7^ Thrombocytopenia is found in 35% – 45% of patients, and neutropenia in about 40%. The symptoms and signs associated with MDS are related to the cytopenia and vary in severity. They can include pallor, fatigue, dyspnoea, palpitations, fever, recurrent infections, bruising and abnormal bleeding.^7^ In more extreme cases of thrombocytopenia, gastrointestinal bleeding or intracranial haemorrhage may occur.

**FIGURE 1:**
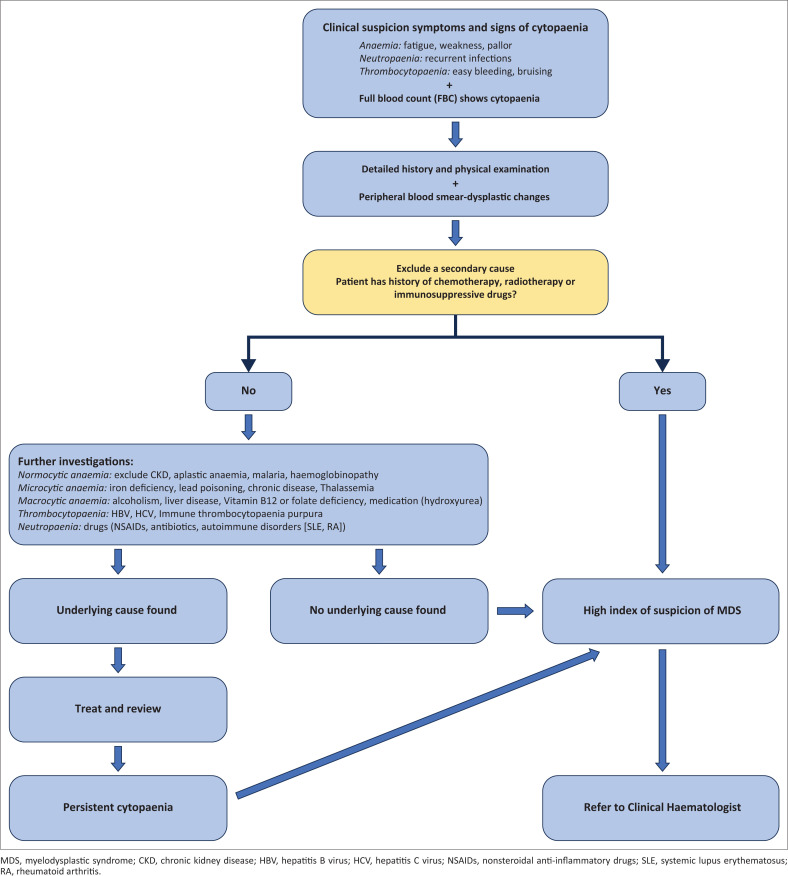
Diagnostic approach to myelodysplastic syndromes for primary care providers.

It can be difficult to suspect MDS in patients presenting with anaemia alone as there is a high prevalence of anaemia in South Africa because of nutritional deficiencies and other diseases, notably human immunodeficiency virus (HIV) and TB.^8,9^ A systematic approach should be adopted to identify the specific cause of the cytopenia and refer those individuals with high risk of MDS. Furthermore, MDS should be always considered in the differential diagnosis of any older adult with unexplained or persistent cytopenia (lasting six months or longer). Figure 1 outlines a practical approach for primary care providers to diagnose MDS.

History taking in a patient with cytopenia (with or without symptoms and signs) should include an inquiry into occupation, medical conditions, medications and alcohol use. The presence of pallor, purpura, petechiae, hepatomegaly, splenomegaly or lymphadenopathy on physical examination should prompt further investigations. These could include the following:

- *A full blood count with differential and reticulocyte count*
- *Peripheral blood smear:* A peripheral blood smear is a non-invasive and cost-effective investigation as it provides information about the morphology of blood cells that can suggest the presence of MDS.^7^ The presence of circulating blasts or cell dysplasia in a peripheral smear should be discussed with a haematologist.
- *Vitamin B12 and folate:* In patients with macrocytic anaemia.
- *Iron studies:* In patients with microcytic anaemia
- *Thyroid function tests*
- *Liver function tests*
- *Urea and electrolytes, renal function tests*
- *Screening for infections:* HIV, hepatitis B and C, malaria
- *Faecal occult blood test:* Screening for occult blood loss
- *Bone marrow biopsy:* The definitive diagnostic test for MDS is a bone marrow biopsy. The presence of hypercellular (or hypocellular) bone marrow with dysplasia is suggestive of MDS. An excess of marrow immature cells (blasts) may also be present. Histological examination can also exclude other causes of cytopenia.^10^
- *Specialist investigations:* Further investigations by a haematologist can include molecular analysis and flow cytometry of blood and marrow cells to confirm the diagnosis.^10^

## Prognosis of individuals with myelodysplastic syndromes

The prognosis of patients with MDS varies greatly and is predicted using validated scoring systems such as the International Prognostic Scoring System (IPSS).^11^ Factors such as the percentage of blasts, karyotype and number of cytopenias influence the prognosis.^12^ Advancements in molecular methods, such as next-generation sequencing (NGS), have enabled the integration of molecular data into risk stratification systems, that is IPSS-M, thereby enhancing the prognostic capabilities of traditional scoring systems.^7^ Those with low-risk MDS have a median survival of three to ten years, while individuals with high-risk disease have a median survival of less than three years.

## Management of myelodysplastic syndromes

### Conservative management

All patients with MDS should be discussed and evaluated by a haematologist before deciding on treatment options. Elderly, asymptomatic patients with chronic mild cytopenias can be managed conservatively with regular follow-up and non-invasive therapy. Conservative treatment is also indicated for those with comorbidities who would not benefit from chemotherapy. Modalities of treatment include erythropoiesis stimulating agents, such as erythropoietin, blood transfusions, growth factors and lenalidomide (an immunomodulatory drug). Erythropoietin can be used to stimulate red blood cell production and can improve anaemia in 15% – 40% of patients for about eight to 23 months.^13^ Blood transfusions should ideally be done with leucodepleted red cells to delay sensitisation as these patients require frequent transfusions.

Supportive care is an important aspect of MDS management and entails observation, clinical monitoring, psychosocial support and quality-of-life (QoL) assessment. Patient education and support is a vital part of management and special interest bodies such as the MDS-Foundation provide patients and communities with relevant information and resources.^14^ Stem cell transplants are not recommended for patients with lower-risk MDS, as the risks associated with treatment may outweigh those posed by the disease.

## Management of higher-risk myelodysplastic syndromes

First-line treatment for those with higher-risk MDS is with hypomethylating agents (such as azacitidine, decitabine or decitabine/cedazuridine) and, if possible, stem-cell transplantation. Haematopoietic stem cell (HSC) transplant is currently the only potentially curative treatment for MDS. However, less than 10% of affected individuals receive this treatment because of limiting factors which include patient age, comorbidities and donor availability. Recent advancements in haematological oncology aim to optimise transplant outcomes by improving patient selection, employing targeted therapies to achieve deeper molecular responses, developing conditioning regimens with reduced toxicity, enhancing molecular tools for early relapse detection and incorporating maintenance strategies post-transplant.^2,13,15^

## Acute myeloid leukaemia

The diagnosis of AML is made when there are at least 20% myeloid blasts (immature cells) in the blood or bone marrow and should be managed by a specialist clinical haematologist.

The goals of treatment for AML are to eradicate the myeloid blasts to the lowest level possible (remission), usually <5% blasts in the bone marrow. The only possible cure is stem cell transplantation.

## Haematopoietic stem cell transplantation

The long-term survival rate following a stem cell transplant ranges from 40% to 50%. However, outcomes depend on factors such as the patient’s age, prognostic scores and donor characteristics. The procedure involves administering high-dose chemotherapy, and in some cases radiation, followed by an infusion of stem cells.

Haematopoietic stem cells that are progenitor cells can be derived from three main sources:

- *Bone marrow*: Cells are extracted from the bone marrow of a donor
- *Peripheral blood (PBSC)*: Cells are collected from the bloodstream of a donor
- *Umbilical cord blood*: Cells are collected from the umbilical cord of a newborn

However, peripheral blood stem cells (PBSC) are the most common source of stem cells and are used in over 80% of adult haematopoietic stem cell transplantation (HSCT) worldwide.

### Unrelated donor registries

The two stem cell registries are the German Bone Marrow Donor (Deutsche Knochenmarkspenderdatei, [DKMS]) and the South African Bone Marrow registry (SABMR), which are non-profit organisations. DKMS maintains a donor registry spanning seven countries with over 12.2 million donors and 114 000 donations.^10^ However, South African patients are underrepresented in global donor databases because of unique HLA (Human Leucocyte Antigen) characteristics, making it difficult to find matches. DKMS Africa is working to increase awareness to recruit donors and increase stem cell donations.

### Challenges in South Africa

Less than 1% of the South African population includes registered volunteer blood stem cell donors, a significantly lower rate compared to other countries like the United States. Recent advocacy campaigns aim to address this disparity by expanding the donor registry. The diagnosis of MDS in an eminent member of the medical profession highlighted the urgent need to expand the donor registry in South Africa and sparked a campaign to register 1000 new donors to the DKMS registry in 2024.^16^

### Eligibility criteria for stem cell transplant

To qualify for a stem cell transplant, patients must be under 70-years-old and have adequate cardiovascular, liver and renal function. Patients must also be physically active and able to perform daily activities independently. They will also require a reliable caregiver during and after the transplant.

### Donor search

The donor can be a sibling, parent or an unrelated individual with matching HLA characteristics. Unfortunately, only one-third of patients have a family member who is a suitable match. In others, a search is conducted for unrelated donors in the stem cell registries. Blood samples of the patient are sent for HLA typing to specialised laboratories, such as the Tissue Typing Laboratory at the National Health Laboratory Service (NHLS) in Cape Town or the South African National Blood Services (SANBS).

### Screening for transplant

A comprehensive evaluation is conducted of the patient’s health and suitability for transplant. Once a matching donor is located, the patient is screened for infectious diseases (e.g. HIV, cytomegalovirus, Herpes simplex toxoplasmosis, Hepatitis B and C, Epstein-Barr virus, malaria, syphilis), ABO blood typing, full blood count (FBC), bleeding time and pregnancy testing (in females).

Before the transplant, disease-modifying therapies can be used to optimise control of MDS. Patients receive myeloablative chemotherapy (high-dose chemotherapy) to eradicate the residual blasts and prepare the bone marrow for the transplant.

## Stem cell infusion and engraftment

The transplant day, referred to as ‘Day 0’, involves infusing the harvested stem cells through a central venous line. The procedure may take 1–2 h to complete.

Engraftment is the process by which transplanted HSCs travel to and settle in the bone marrow and begin producing new blood cells (red blood cells, white blood cells and platelets).^17^ Several factors contribute to successful engraftment including the physical condition of the patient, severity of disease, age of patient, type of donor and the HSCT conditioning regimen.^15^ It is a key milestone in the recovery process after a stem cell transplant. Engraftment usually occurs within 2–4 weeks after a stem cell transplant. White blood cells are usually the first to engraft, followed by red blood cells and platelets.

## Management of transplant side effects

While HSCT can be lifesaving, it is associated with risks and potential side effects, both short-term and long-term.^17^ A summary of short-term and long-term complications is provided in Table 1.

- *Graft-versus-host disease* (GVHD): Graft-versus-host disease occurs when the donor’s immune cells attack the recipient’s tissues. Acute GVHD typically occurs within the first 100 days post-transplant, affecting the skin (rash, redness), liver (jaundice) and gastrointestinal tract (diarrhoea, abdominal pain). Chronic GVHD starts more than 3 months after transplantation causing symptoms like dry eyes, joint stiffness, skin thickening and organ dysfunction.
- *Short-term side-effects*: Immune suppression because of conditioning chemotherapy, radiation and immunosuppressive medications increases the susceptibility to bacterial, viral and fungal infections. Painful inflammation and ulceration of the mouth and gastrointestinal tract can occur and may result in increased risk of infection and difficulty eating. This may result in the patient requiring total parenteral nutrition (TPN). Patients may also experience profound tiredness because of intensive conditioning therapy and recovery demands. Nausea and vomiting can occur as a result of chemotherapy or radiation used in conditioning regimens. Hair loss is another common side effect of the chemotherapy or radiation. Cytopaenias may also occur because of the myeloablative conditioning regimen which causes depletion of blood cells before the transplanted stem cells engraft and begin producing new ones. Conditioning regimens may cause damage to multiple organs including the liver (sinusoidal obstructive syndrome/veno-occlusive disease), kidneys, lungs or heart.
- *Long-term side effects*: Patients who have undergone HSCT are at an increased risk of developing secondary cancers later in life because of prior exposure to chemotherapy and radiation. Endocrine problems such as hypothyroidism or infertility can occur because of radiation and chemotherapy. Osteoporosis or avascular necrosis may result from long-term steroid use, and pulmonary fibrosis or bronchiolitis obliterans may occur because of GVHD or prior treatment. Patients who have undergone HSCT for a haematological malignancy may have a recurrence of the underlying disease being treated with the transplant.
- *Psychological effects*: The emotional and physical toll of the transplant process and the prolonged isolation required during the transplant and recovery period may contribute to patients experiencing depression, anxiety and cognitive issues.

**TABLE 1:** Short and long-term side-effects following stem cell transplant.

Short-term side-effects	Long-term side-effects
Graft-versus-host disease (GVHD) Opportunistic infections Mucositis Fatigue Nausea and vomiting Hair loss Organ toxicities Anaemia, thrombocytopaenia and neutropenia	Chronic Graft-versus-host disease (GVHD) Secondary cancer Endocrine problems Bone and joint problems Pulmonary complications Relapse Psychological effects

## Care of patients after stem cell transplant

In the first three months patients should be monitored for complications and strict infection prevention strategies should be followed. Any side-effects can be managed symptomatically.

After three months, patients will need to be reviewed regularly for long-term complications, such as damage to organs like the liver, kidney, lungs and heart. Other potential issues include cataracts, bone and muscle weakness and problems with hormone production, such as an underactive thyroid or type 1 diabetes, and new malignancies. An essential aspect of care is to offer mental health support and address physical and emotional challenges.

Primary care providers need to work with specialists to ensure smooth transition of care before, during and after transplant.

## Conclusion

Primary care providers should consider MDS, especially in older individuals who present with cytopaenias. Modalities of treatment include disease-modifying therapies, chemotherapy and radiotherapy. Stem cell transplantation is the only possible cure for higher-risk MDS and AML. However, there is a need for increased awareness and drive to improve the donor registry.
